# Poetry

**Published:** 1870-07

**Authors:** 


### POETRY,

### AND ALL ABOUT AN OLD MISER.

" A cautious look around he stole,
His bags of chink he chunk;
And many a wicked smile he smole,
And many a wink he wunk."

Sometimes it is desirable to give medicine unawares, and let the patient find it out afterwards; so in quoting the above we have administered a pill of laugh; for whoever could refrain from a loud gulph on reading the miser's lines, hasn't as much fun in him as the little urchin, who, seeing a little mouse come down the bell-rope and commence tugging at the school-master's gown, while kneeling at prayer, burst out into an uncontrollable fit of laughter. The domine was greatly put out, and threatened dire punishment unless the little fellow could explain himself in poetry, whereupon he impromptued the following rhyme, —

" A little mouse, for want of stairs,
Came down the rope to say his prayers."

The same little boy afterwards became ISAAC WATTS.

